# Overview of Breastfeeding Under COVID-19 Pandemic

**DOI:** 10.3389/fimmu.2022.896068

**Published:** 2022-05-31

**Authors:** Zehan Pang, Ruolan Hu, Lili Tian, Fuxing Lou, Yangzhen Chen, Shuqi Wang, Shiting He, Shaozhou Zhu, Xiaoping An, Lihua Song, Feitong Liu, Yigang Tong, Huahao Fan

**Affiliations:** ^1^ College of Life Science and Technology, Beijing University of Chemical Technology, Beijing, China; ^2^ Health & Happiness Group, Health & Happiness Research, China Aesearch and Innovation, Guangzhou, China

**Keywords:** SARS-CoV-2, COVID-19, breastfeeding, vertical transmission, human milk, lactoferrin

## Abstract

During the global pandemic of coronavirus disease 2019 (COVID-19) caused by severe acute respiratory syndrome coronavirus 2 (SARS-CoV-2), pregnant and lactating women are at higher risk of infection. The potential of viral intrauterine transmission and vertical transmission by breastfeeding has raised wide concerns. Breastmilk is rich in nutrients that contribute to infant growth and development, and reduce the incidence rate of infant illness and death, as well as inhibit pathogens significantly, and protect infants from infection. Although it is controversial whether mothers infected with COVID-19 should continue to breastfeed, many countries and international organizations have provided recommendations and guidance for breastfeeding. This review presents the risks and benefits of breastfeeding for mothers infected with COVID-19, and the reasons for the absence of SARS-CoV-2 active virus in human milk. In addition, the antiviral mechanisms of nutrients in breastmilk, the levels of SARS-CoV-2 specific antibodies in breastmilk from COVID-19 infected mothers and vaccinated mothers are also summarized and discussed, aiming to provide some support and recommendations for both lactating mothers and infants to better deal with the COVID-19 pandemic.

## 1 Introduction

During the global pandemic of SARS-CoV-2, there were considerable controversies about whether mothers infected with COVID-19 should adhere to breastfeeding, which is beneficial to infant growth and development (1). However, the potential risks of infection with SARS-CoV-2 in infancy during breastfeeding also need to be considered (2). In addition, there are limited clinical trials that have been conducted on the vaccination of lactating women (3, 4), and some studies have shown that immunogenicity can protect mothers and infants from COVID-19 infection (3, 5).

Breastmilk is rich in nutrients (6, 7), bioactive molecules (cytokines, immunoglobulins (Igs), growth factors, and immune cells) (8, 9), antibodies (6), and microorganisms (8), which contribute to infants for their growth and development, maturation of immune, development of organs, and microbial colonization (6). The active and passive immunity provided by breastmilk plays an important role in strengthening the infant’s response to infectious diseases (10). Breastfeeding in the first few months of life has been shown to reduce the incidence of infections and illness (1).

Breastmilk serves different purposes at various stages during pregnancy. At the end of the mother’s pregnancy, the mammary gland produces colostrum, which is rich in bioactive factors providing passive immunity to the newborn (8, 11–13), and preventing chronic immune-mediated diseases for a long time after weaning (8). 15 to 90 days after delivery, mature milk is produced in the mammary glands (8). Compared to colostrum, mature milk has a lower content of Igs (14, 15), proteins (14), cytokines (8, 14), a higher content of lipidic compositions (15), and carbohydrates (16) which can provide more energy for the infants (16). Large amounts of Igs in the mother’s body can be transmitted to the fetus or infant through the placenta or breastmilk (17). After full pregnancy, the immune system of the newborn is still immature, breastmilk can act as an exogenous factor to stimulate the development of its mucosal immune system, and the infants can also develop active immunity to antigens contained in breastmilk. During the period with an immature system, infants rely on the exogenous protection of breastmilk, while breastmilk also promotes the maturation of their endogenous mucosal system (8).

The specific antibodies against SARS-CoV-2 have been proved in breastmilk from infected or vaccinated [BNT162b2 (18, 19), mRNA-1273 (19), ChAdOx1-S (20), CoronaVac (21)] mothers (22, 23). Interestingly, specific antibodies are also found in neonatal umbilical cord blood and saliva of breastfed infants (11, 24, 25), proving the antibodies could be transferred to and protect the infants without significant adverse effects, which could explain the resistance of some neonates to SARS-CoV-2 (12). Moreover, anti SARS-CoV-2 secretory immunoglobulin A (IgA) can passively protect neonates (5, 12, 22), while the IgA-spike antigen immune complex can also actively stimulate and strengthen the autoimmune system of newborns (12). Since there are still a few clinical trials on vaccination for lactating women and the relevant results may be associative (4), it is suggested that lactating women should receive the vaccine after considering their situation and consulting with doctors.

In addition, SARS-CoV-2 RNA could be detected in the milk of COVID-19-infected mothers (26), but no infectious viral particles could be isolated (27). Thus, there is no clear evidence that breastmilk can transmit SARS-CoV-2 (28). The specific SARS-CoV-2 antibodies contained in the breastmilk of mothers infected with COVID-19 can protect infants against COVID-19 infection (29). These findings suggest that the benefits of breastfeeding outweigh the risks. However, the transmission of SARS-CoV-2 through respiratory droplets during breastfeeding cannot be excluded according to the current researches (30). Therefore, mothers infected with COVID-19 should take effective protective measures while breastfeeding, including wearing a medical mask, disinfecting the hands with 70% ethanol, and wiping the breast (31, 32).

This review will analyze the possibility of vertical transmission of breastfeeding and summarize the reasons for the absence of infectious SARS-CoV-2 virus in breastmilk. Moreover, the situation of SARS-CoV-2 specific antibodies existing in the breastmilk of COVID-19 infected patients and COVID-19 vaccinated mothers were systematically summarized.

## 2 Low Risk of Vertical Transmission

Due to the vulnerable immune system of newborns, many clinicians and researchers are concerned about the risk of vertical transmission of SARS-CoV-2 from COVID-19-infected mothers to the fetus or newborn. Neonatal infected COVID-19 is uncommon, and the majority of infants with positive test results for SARS‐CoV‐2 are asymptomatic (29, 33). In a few studies, infants infected with COVID-19 had a benign course, and mild symptoms (33, 34), however, intrauterine virus exposure may affect fetal health and ultimately affect the outcomes of pregnancy (35, 36).

Vertical transmission is referred to mother-to-child transmission of pathogens during antepartum and intrapartum, or to the newborns through the placenta, body fluid contact during birth, or through direct contact during breastfeeding in the postpartum period (37). The currently proposed mechanism of vertical transmission has focused on placental transmission (38). As an entry receptor of SARS-CoV-2, angiotensin-converting enzyme 2 (ACE2) is highly expressed in cells at the maternal-fetal interface (including decidual stromal cells, perivascular cells, and cytotrophoblast and syncytiotrophoblast in the placenta) (36, 39). Therefore, the placenta can be infected with SARS-CoV-2 theoretically (36). However, the current study suggests that the probability of placental transmission is usually less than 5% (36). Jafari M et al. (33) revealed that SARS-CoV-2 RNA was detected in 12% of placental specimens, 5.6% of amniotic fluid, and 6% of umbilical cord swabs, indicating a low rate of presence of virus in the placenta. According to the data analysis of Sweeney I et al. (40), 17 of 184 placental samples were positive for SARS-CoV-2 RNA, of which 7 cases were detected SARS-CoV-2 in the maternal, neonatal, and placental tissue (41), suggesting a low risk of placental transmission. It’s worth noting that cesarean delivery would not significantly reduce the risk of vertical transmission, on account of increasing placental permeability by placental cells with high ACE2 expression, where the virus can invade and destroy the placenta (33).

In addition, the detection of SARS-CoV-2 RNA in the breastmilk of mothers infected with COVID-19 (26) has raised concerns about the transmission of the SARS-CoV-2 through breastmilk (42). Current studies have demonstrated that the virus cannot enter the mammary gland (43), and no live SARS-CoV-2 has been isolated in breastmilk (29, 43), making it unlikely that breastmilk is a vector for SARS-CoV-2 transmission (44).

## 3 Lack of Breastmilk Causes Illness and Even Death in Newborns

Breastmilk is rich in nutrients, including Igs, lactoferrin, human milk oligosaccharides, and anti-inflammatory factors (45) which are beneficial for newborns, while partial breastfeeding or non-breastfeeding increases the risk of neonatal diarrhea or respiratory infections and decreases infant survival rate (1, 46). Newborns or preterm infants lacking breastfeeding are prone to Sudden Infant Death Syndrome, necrotizing enterocolitis (NEC) and sepsis (1), and even higher mortality than breastfed infants (27). Thus, refusing breastfeeding to preterm infants due to the potential risk of COVID-19 vertical transmission may lead to more serious consequences.

Moreover, the anti-pathogen infection and immunological benefits of breastfeeding are well established (1). Breastmilk contains antibodies, free fatty acids, lactoferrin, milk fat globule membrane (MFGM), human milk oligosaccharides (HMO), and other antiviral ingredients (42, 47, 48). These nutrients can bind to viral receptors, limiting the ability of viral entry to achieve antiviral activity (47).

Lactoferrin has now been shown to inhibit viruses such as herpes simplex virus, rotavirus, coronavirus, and cytomegalovirus, and also has antibacterial, antiparasitic, and antifungal effects (47, 49, 50). MFGMs contain a variety of glycosylated proteins and lipids that have been demonstrated the inhibition of rotavirus binding to cell membranes significantly (47, 51). HMO inhibits the activity of norovirus, rotavirus, and influenza virus by balancing cytokine response, stimulating the maturation of epithelial cells, and reducing viral adherence to target cells (47). It can also inhibit many Gram-negative pathogenic bacteria such as *Klebsiella pneumoniae*, *Acinetobacter baumannii*, *Pseudomonas aeruginosa*, and *Burkholderia cenocepacia*, and many Gram-positive bacteria such as *Staphylococcus aureus*, *Enterococcus faecium*, and *Enterococcus faecalis* (52). Moreover, it has been demonstrated that Osteopontin can inhibit rotavirus infection *in vivo*, and glycerol monolaurate has been described to have a wide range of microbial inhibitory properties (47).

## 4 SARS-CoV-2 RNA Positive in Breastmilk But Without Infectious Virus Isolated

According to the analysis of Jafari M et al., 5% of breastmilk samples were positive for SARS-CoV-2 RNA (33), but no infectious virus could be isolated (27), which implies that the breastmilk from lactating mothers with COVID-19 is not infective, and there are two possible reasons summarized below.

### 4.1 Low Expression of SARS-CoV-2 Essential Host Factor in Mammary Glands

**Figure 1:**
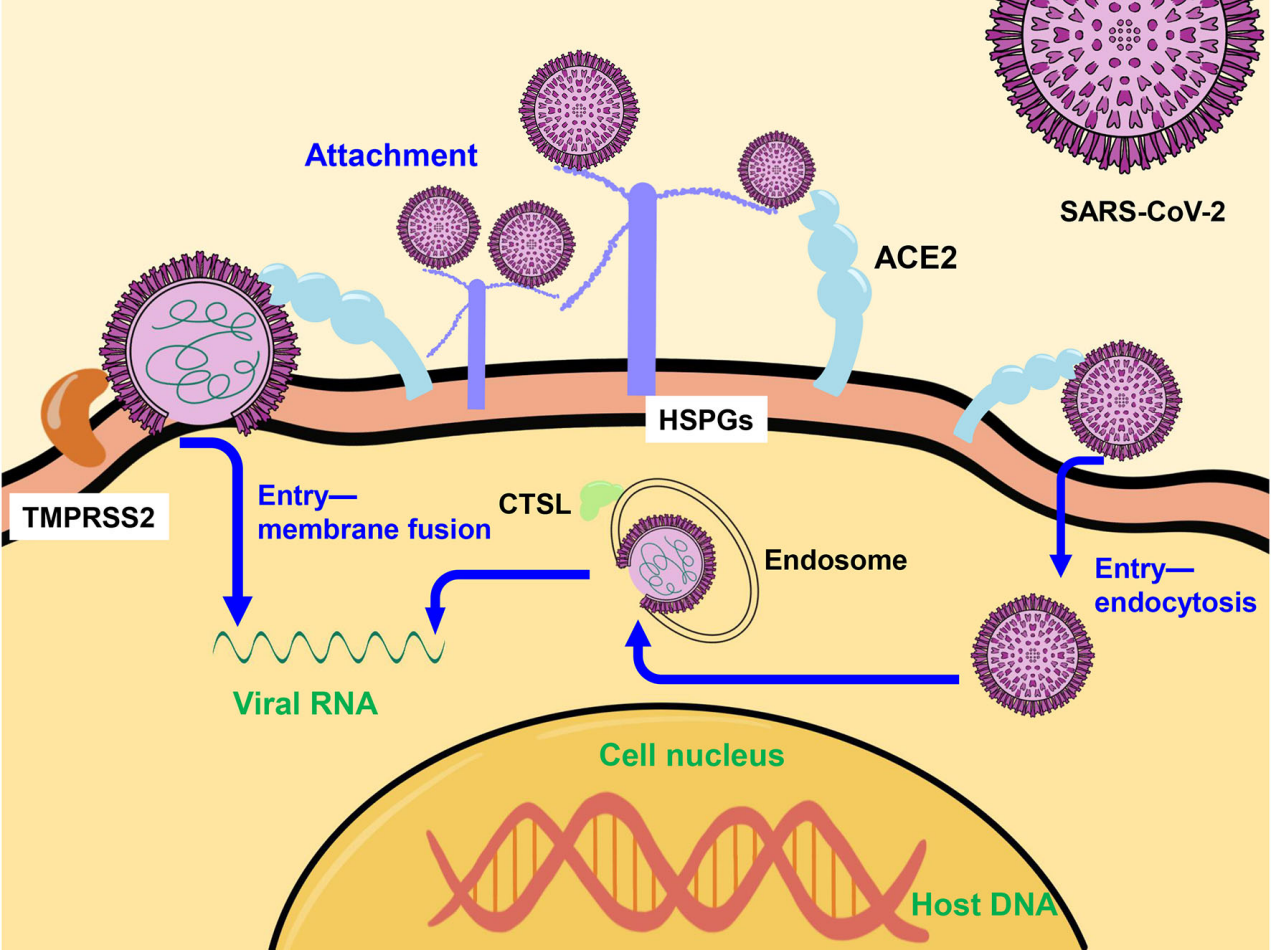
The mechanism of SARS-CoV-2 entry into the host cell. SARS-CoV-2 enters into cells by membrane fusion-mediated with the assistance of TMPRSS2 or *via* the endocytosis route to form endosome, and release genomic RNA into cytoplasm directly with the assistance of CTSL. TMPRSS2, transmembrane protease serines 2; CTSL, cathepsin L.

SARS-CoV-2 binds to heparan sulfate proteoglycans (HSPGs) and is highly enriched in the cells surface (53), and then initiates two entry pathways assisted by high-affinity receptor angiotensin-converting enzyme 2 (ACE2). In cells with high transmembrane protease serines 2 (TMPRSS2) expression (e.g. Calu-3, a human Lung Cancer Cell Line which is with high TMPRSS2 expression but deficiency in cathepsin L), SARS-CoV-2 enter into cells by membrane fusion-mediated infection after TMPRSS2 cleaves S protein into S1 and S2 subunits (54, 55), and binds with S2 subunit (55, 56). In cells with low TMPRSS2 expression (e.g. Vero E6, an African green monkey kidney cell line), SARS-CoV-2 enters into cells *via* the endocytosis route to form endosome, and SARS-CoV-2 genomic RNA are released from endosome into cytoplasm directly with the assistance of cathepsin L (CTSL) (55, 56) (**Figure 1**). And in human primary lung epithelial cells or human colorectal adenocarcinoma epithelial cell line Caco-2, both routes are utilized for SARS-CoV-2 entry (57–59). And ACE2 is expressed at extremely low levels in female reproductive organs and mammary glands (27, 38). Only 5% of mammary glands luminal epithelial cells manufacturing human milk express ACE2, and none of the cells co-express ACE2 with TMPRRS2 or CTSL (27, 43). Consequently, mammary cells are not susceptible to SARS-CoV-2 infection (38).

It was shown that ACE2 expression levels in the mammary tissue of female mice were regulated by cytokines such as JAK/STAT dependent enhancers and prolactin during pregnancy and lactation, with a 13-fold increase of mRNA. In contrast, TMPRSS2 expression was not further induced, suggesting that it is not controlled by the JAK/STAT pathway (60).

Although ACE2 expression may be further induced during lactation, mammary cells lacking TMPRSS2 and CTSL expression are unlikely to have significant amounts of SARS-CoV-2 active virus.

### 4.2 Antiviral Mechanism of Nutrients in Breastmilk Against SARS-CoV-2

With various nutrients, breastmilk can inhibit the activity of SARS-CoV-2, so the infectious SARS-CoV-2 cannot be isolated in breastmilk.

#### 4.2.1 Whey Protein

**Figure 2:**
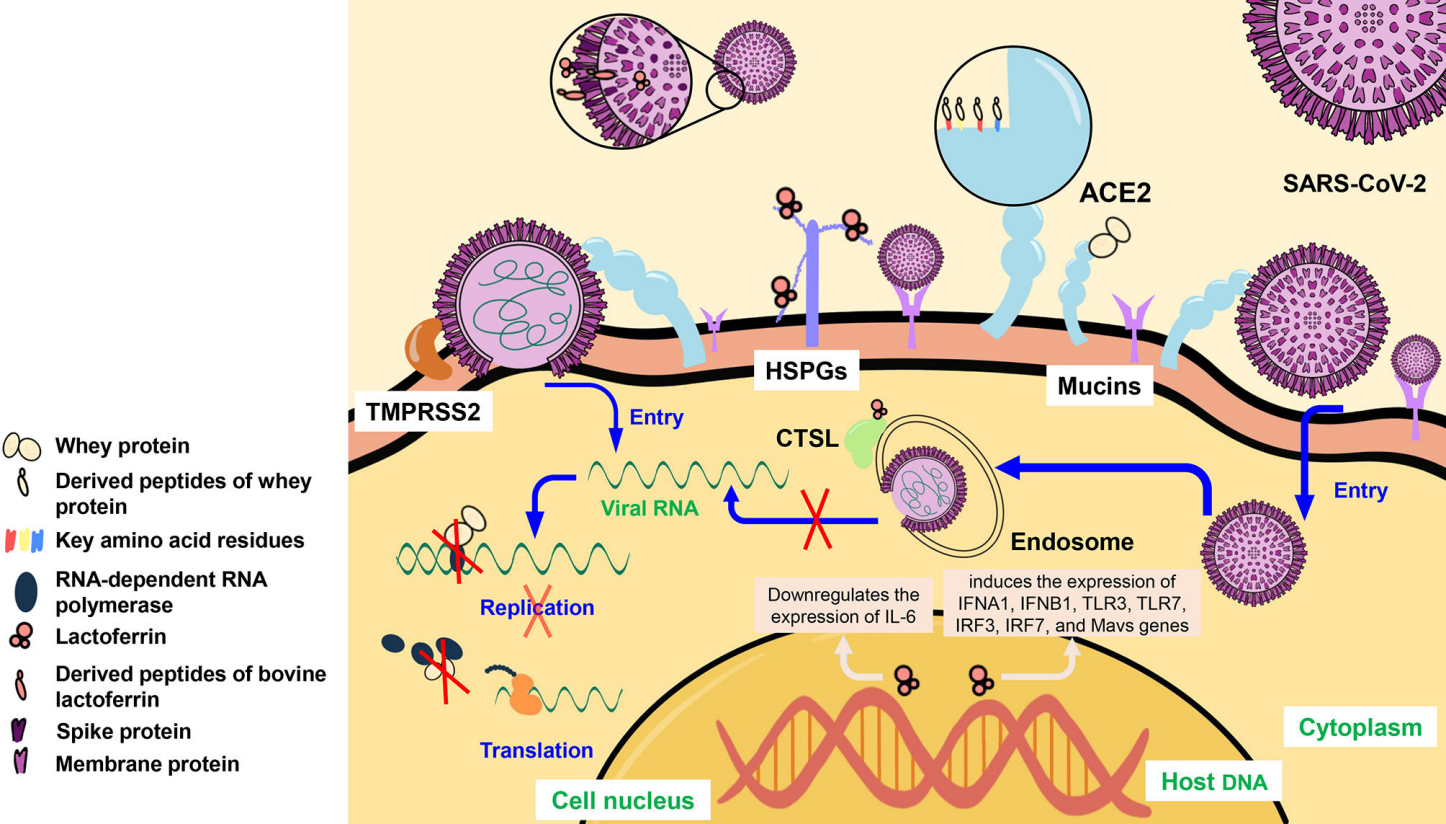
The mechanism of whey protein, lactoferrin, and mucins inhibition of SARS-CoV-2 infection. Whey protein can bind to ACE2 of the host cell and can enter the cell to bind to RdRp, derived peptides of whey protein can bind to key amino acid residues of ACE2. LF can bind to HSPGs and CTSL. Meanwhile, LF can bind to SARS-CoV-2 S proteins and the derived peptide of bLF can bind to SARS-CoV-2 M proteins. In addition, LF entering the host nucleus can induce the expression of IFNA1, IFNB1, TLR3, TLR7, IRF3, IRF7, and MAVS genes, and downregulate the expression of IL-6. Mucins can cover the mucosal cell surface and provide a large number of sialylated residues, and bind to SARS-CoV-2. ACE2, angiotensin-converting enzyme 2; HSPGs, heparan sulfate proteoglycans; TMPRSS2, transmembrane protease serines 2; CTSL, cathepsin L; IL-6, interleukin- 6; IFNA1/IFNB1, interferons A1/B1; TLR3/TLR7, toll-likereceptors 3/7; IRF3/7, interferon regulatory factors 3/7.

Whey protein, which is a mixture of various bioactive components, can inhibit SARS-CoV-2 infection and the production of infectious viral particles by blocking viral entry and replication (61) (**Figure 2**). Whey protein can block the binding of ACE2 and SARS-CoV-2 S protein to inhibit virus entry, and reduce the RNA-dependent RNA polymerase (RdRp) activity of SARS-CoV-2, inhibiting the virus replication at post-entry (61).

Through molecular docking, researchers confirmed that IPP, LIVTQ, IIAE, and LVYPFP of whey protein-derived peptides could interact with the key amino acid residues Glu 375, his 378, Glu 402, and Tyr 515 at the active site of ACE2 through hydrogen bond or/and salt bridge (62), and further verified that whey protein could inhibit the binding of SARS-CoV-2 to ACE2 of the host cell.

#### 4.2.2 Lactoferrin

LF is reported to inhibit SARS-CoV-2 infection (63, 64), and it is a defense molecule on the mucosal surface, the highest content in the human colostrum (64, 65), and can bind to iron and other metals (50, 64). At present, iron overload is considered as a factor in the pathogenesis of COVID-19. As a natural iron chelator, LF can protect patients (66) and can bind to various receptors of SARS-CoV-2 and inhibit the viral entry into host cells (50, 66) (**Figure 2**).

The nanomolar range of LF mediates the activity of anti-SARS-CoV-2 S protein by targeting HSPGs co-receptor *in vitro*, which can prevent the virus from attaching to host cells (50, 53, 67). In addition, as an immune modulator (68), LF significantly induces the expression of interferons (IFNs), including IFNA1 and IFNB1, toll-like receptors (TLRs), including TLR3 and TLR7, interferon regulatory factors (IRFs), including IRF3 and IRF7, and Mavs genes (68), which enhance the interferon response (53, 67). Because of the ability to sequester free iron, LF can mediate the downregulates of proinflammatory cytokine gene expression by entering inside the nucleus of host cells. It can downregulate the expression level of interleukin- 6, and reduce the occurrence of cytokine storms (69–71).

There are three proteins on the membrane of SARS-CoV-2, namely S protein, membrane protein (M protein), and envelope protein (E protein). After silico hydrolysis of bovine lactoferrin (bLF), bLF hydrolytic peptide GSRY with good solubility that can bind to M protein was identified by the molecular docking method (72). The molecular docking also demonstrated that LF can not only bind to CTSL and affect the virus internalization of SARS-CoV-2 (73) but also target SARS-CoV-2 S protein and block its binding to ACE2 (74).

The combination of LF and drugs, such as remdesivir (53), hydroxychloroquine (75), and hypothiocyanite (76), can enhance the antiviral activity against SARS-CoV-2. *Lacticaseibacillus paracasei* DG (77) and *LigiLactobacillus Salivarius* SGL03 (78) can also enhance the antiviral activity against SARS-CoV-2 of LF.

Clinical trials on the efficacy, safety, and tolerability of oral and intranasal liposome bLF in asymptomatic and mild to moderate COVID-19 patients (NCT04475120) have been conducted. The preliminary experimental results of inhibition of SARS-CoV-2 infection showed that liposomal bLF has a better therapeutic effect than bLF only, and can shed viruses, relieve clinical symptoms, reduce the risk of transmission and infection (79). Interestingly, bLF is more effective than human LF (53) to inhibit SARS-CoV-2 infection.

#### 4.2.3 Mucins

Mucins can cover the surface of mucosal cells, providing many sialylated residues that are similar to the residues on the cell membrane and can bind to SARS-CoV-2 and prevent it from entering into cells (80, 81) (**Figure 2**). At the same time, mothers infected with SARS-CoV-2 are speculated to harbor more mucins in their breastmilk and can potentially protect the infants from COVID-19 (81).

#### 4.2.4 Secretory Immunoglobulin A

**Figure 3:**
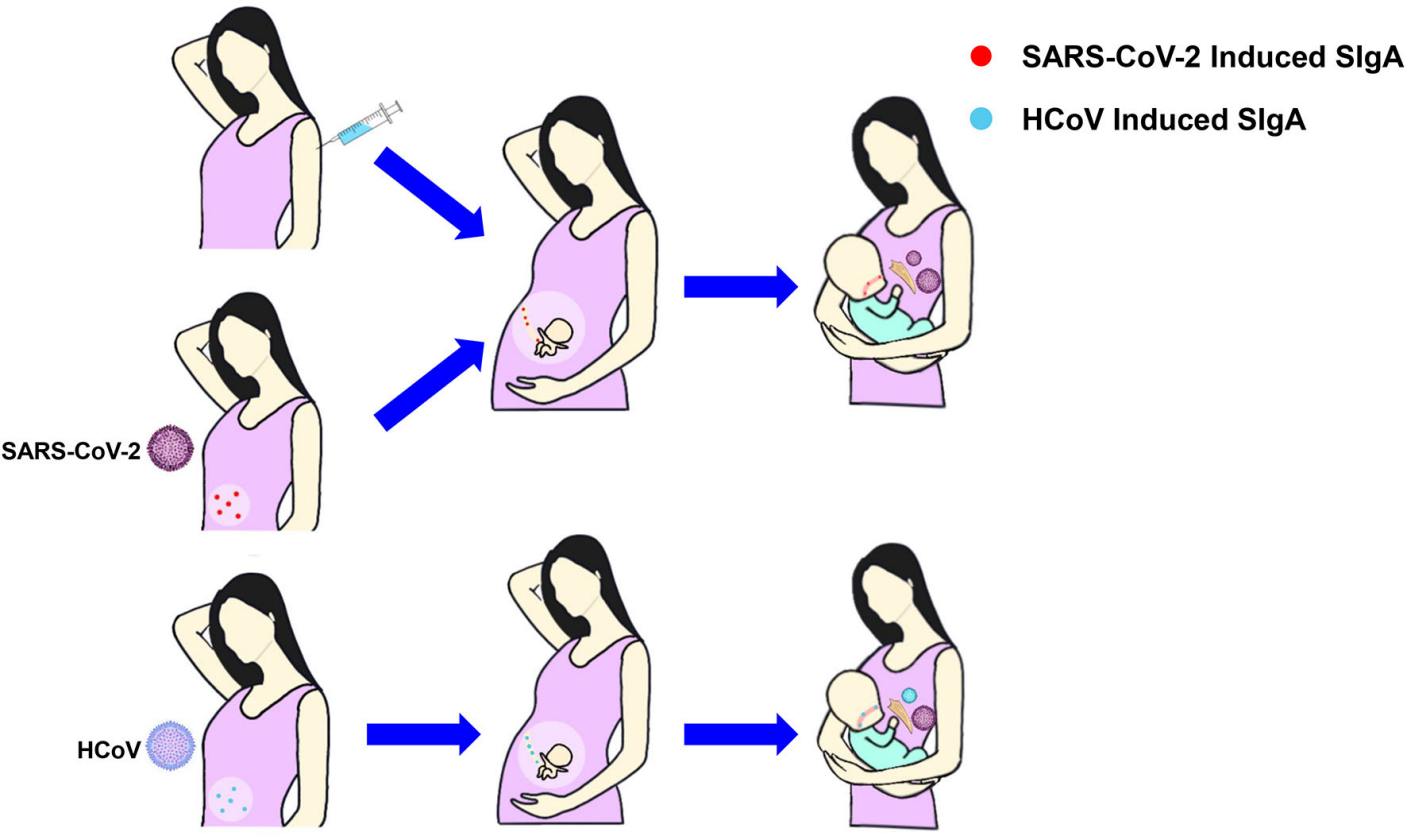
The production and transfer of SIgA in breastmilk. Mothers infected with COVID-19 or vaccinated with COVID-19 have IgA antibodies in breastmilk that can be transferred to the fetuses through the placenta or to the infants through breastmilk, protecting them from infection. SIgA induced by HCoV can cross-immunize SARS-CoV-2. SIgA, Secretory Immunoglobulin A; HCoV, Human coronavirus.

Mothers infected with COVID-19 or vaccinated with COVID-19 have IgA antibodies in breastmilk, which is also considered to be one of the reasons for the absence of the SARS-CoV-2 infectious virus in breastmilk (82). Human coronavirus (HCoV) induced SIgA antibody in breastmilk can provide cross-immunity to SARS-CoV-2 (83, 84), and SIgA contained in pre-pandemic breastmilk samples is capable of cross-reacting with SARS-CoV-2 (85). What’s more, IgA antibodies can be transferred to infants through breastmilk, further providing infants with immune protection against COVID-19 (86) (**Figure 3**).

### 4.3 Pasteurization Can Inactivate Potential Infectious SARS-CoV-2 in Breastmilk

Using pasteurization to heat at 62.5°C for 30 minutes can inactivate bacteria and viruses, including SARS-CoV-2 (87, 88), while retaining a lot of nutrients and protective effects of breastmilk (42, 89). Compared to formula milk which lacks many important components of breastmilk (42), pasteurized breastmilk is safe and reassured for breastfeeding (29, 90). Some experimental studies have demonstrated that pasteurization does not reduce the level of IgA in breastmilk significantly but diminishes the neutralizing ability of antibodies (27, 91), while pasteurization can preserve the neutralizing capacity of SARS-CoV-2 specific IgA in breastmilk (13, 91).

## 5 Specific Antibodies to SARS-CoV-2 in Human Milk

### 5.1 Antibodies in the Human Milk From Vaccinated People

Previous studies showed that the IgG and IgA levels in breastmilk were high within six months after giving pertussis and influenza vaccination. In this case, the incidence rate of respiratory diseases in the infants also decreased significantly, which suggested that specific antibodies produced in the breastmilk could be transferred to the infants and protect them from being infected after vaccination (18). Similarly, antibodies produced in mothers with the COVID-19 vaccine or infected with COVID-19 can transfer protective antibodies to fetuses or newborns through the placenta or breastmilk, thus protecting fetuses and newborns (92, 93) (**Table 1**).

**Table 1:** Specific antibodies to the SARS-CoV-2 exist in the human milk.

Vaccination or infection	Numbers of samples	Sampled time points (T)	Results	Reference
33 BNT162B2 vaccinators	93 serum and breastmilk samples	T1: two weeks after the first dose T2: two weeks after the second dose T3: four weeks after the second dose	After the second dose, the level of the anti-SARS-CoV-2 S1 IgG antibody in breastmilk increased, which was positively correlated with the corresponding antibody level in serum.	Esteve Palau E et al. (2)
14 BNT162B2 vaccinators	66 breastmilk samples	T1: pre-vaccination T2: 1-3 days after the first dose T3: 7-10 days after the first dose T4: 3-7 days after the second dose T5: 4-6 weeks after the second dose	At T4, the IgA level of anti-spike and anti-RBD in samples was higher than previous time points and samples of convalescent mothers. At T5, the level of IgA decreased but was higher than that at T1.	Low JM et al. (94)
48 mRNA-1273 (21) or BNT162b2 (27) vaccinators	Not mentioned	T1: before the first dose T2: before the second dose T3: 4-10 weeks after the second dose	Samples from vaccinators contained titers of anti-SARS-CoV-2 IgG higher than the convalescent samples. There was no significant difference in the level of anti-RBD IgG in the samples from mothers immunized with mRNA-1273 or BNT162b2 vaccine	Golan Y et al. (19)
23 mRNA-1273 or BNT162b2 vaccinators	46 breastmilk samples	T1: after the first dose T2: after the second dose	10 days after the first dose, 95.65% (22/23) samples contained anti-spike antibodies, 9.09% (2/22) contained IgA, IgG, and IgM, 77.27% (17/22) contained IgA and IgG, and 13.63% (3/22) contained IgA.	Gonçalves J et al. (95)
110 mRNA-1273 (70) or BNT162b2 (20) or one dose ChAdOx1-S (20) vaccinators	Not mentioned	30 days after the second dose of BNT162b2 or mRNA-1273 30 days after the first dose of ChAdOx1-S	IgA and anti-S1 IgG antibodies were contained in all samples, while the level of IgA antibody in the samples from mothers with BNT162b2 or mRNA-1273 was higher than that from mothers vaccinated with ChAdOx1-S.	Lechosa Muñiz C et al (20)
20 CoronaVac vaccinators	170 breastmilk samples	T1: before vaccination T2-4: 7 samples per week for three weeks after the second dose T5: 10 samples were collected four months after the first dose	The level of anti-SARS-CoV-2 IgA increased in the first two weeks of the first dose and increased significantly in the fifth and sixth weeks. At the seventh week, the level of specific IgA antibodies in samples of 10 (10/20) mothers was higher than the serum transformation value. Four months after the first dose, the specific IgA antibodies level in the samples of 5 mothers (5/10) was higher than the serum transformation value at that time.	Calil V et al. (21)
18 infected patients	37 breastmilk samples	6 samples were sampled before the onset or within a week of the symptoms	76% (26/34) of the samples contained SARS-CoV-2 specific IgA, 80% (22/27) of the samples contained SARS-CoV-2 specific IgG.	Pace RM et al. (96)
64 infected patients	316 breastmilk samples	Relative to the day of testing for COVID-19: T1: 78 samples were collected from 40 mothers within a week T2: 120 samples were collected from 58 mothers between day 8 and 21 T3: 89 samples were collected from 47 mothers between day 22 and 57 T3: 89 samples were collected from 47 mothers between day 22 and 56 T4: 29 samples were collected from 29 mothers between day 57 and 106	75% of the breastmilk samples contained anti-RBD IgA and 77% of the breastmilk samples had IgA that persisted for two months.	Pace RM et al. (82)
1 infected patient	2 breastmilk samples	T1: July 2020 T2: October 2020 (6.5 months after infection)	Neutralizing IgG and IgA antibodies in breastmilk remained positive for 6.5 months after infection. Pasteurization reduced the neutralizing capability of breastmilk contained antibodies	Favara DM et al. (89)
60 infected patients and/or recovered patients 13 women before the pandemic	73 breastmilk samples	60 samples were collected during the pandemic 13 samples were collected before the pandemic	82.9% of the samples had at least one antibody, 52.9% of the samples had IgM, IgG, and IgA antibodies. The positive rate of IgG continued to increase while IgA was relatively stable.	Bäuerl C et al. (97)
8 recovered patients and 7 suspected patients	15 breastmilk samples	Not mentioned in detail	All samples presented significant specific IgA antibodies, while 80% of the samples showed anti-RBD IgA activity and 67% showed anti-RBD IgG and/or IgM activity.	Fox A et al. (98)

IgA, Immunoglobulin A; IgG, Immunoglobulin G; IgM, Immunoglobulin M; RBD, Receptor binding domain.

At present, a variety of COVID-19 vaccines have been successfully developed. In the current researches, the vaccines inoculated by lactating mothers are mainly BNT162B2 (18, 19), mRNA-1273 (19), ChAdOx1-S (20), and CoronaVac (21), and there is no adverse reaction in infants has been caused by these vaccines through breastmilk (99).

Several studies have reported the level of antibodies produced in breastmilk from lactating women after inoculating mRNA vaccines (BNT162B2 and mRNA-1273). Esteve-Palau E et al. (2) collected 93 serum and breastmilk samples from 33 BNT162B2 vaccinators, which were taken at 3 points in time: two weeks after the first dose of vaccine, two weeks after the second dose of vaccine, and four weeks after the second dose of vaccine, respectively. The results showed that after the second dose, the level of the anti-SARS-CoV-2 S1 IgG antibody in breastmilk increased, which was positively correlated with the corresponding antibody level in serum. Low JM et al. (94) collected 66 breastmilk samples from 14 lactating women inoculated with BNT162b2 sampled at pre-vaccination, 1-3 days after the first dose, 7-10 days after the first dose, 3-7 days after the second dose, and 4-6 weeks after the second dose, respectively. At the fourth time point, the IgA levels of anti-spike and anti-receptor-binding domain (anti-RBD) in breastmilk were significantly higher than those at the previous time points and also higher than in the breastmilk samples of convalescent mothers with COVID-19. At the fifth time point, the level of IgA decreased. At both the fourth and fifth time point, the content of IgG targeting RBD and spike in breastmilk were significantly higher than that at the first time point. The study of Golan Y et al. (19) included 48 lactating mothers vaccinated with mRNA-1273 (21/48) or BNT162b2 (27/48). They collected blood and breastmilk samples before the first vaccination, before the second vaccination, and 4-10 weeks after the second vaccination, respectively. The results showed that breastmilk contained high titers of anti-SARS-CoV-2 IgG after immunization, which were higher than the convalescent samples after SARS-CoV-2 infection. There was no significant difference in the level of anti-RBD IgG in the breastmilk of mothers immunized with mRNA-1273 or BNT162b2 vaccine. Gonçalves J et al. (95) collected 23 pairs of breastmilk samples before mRNA-1273 or BNT162b2 vaccination and after the first and second vaccination. The results showed that 10 days after the first injection, 22 of the 23 lactating women produced anti-spike antibodies in their breastmilk, 2 of which produced IgA, IgG, and IgM, 17 produced IgA and IgG, and 3 produced IgA.

Meanwhile, Lechosa-Muñiz C et al. (20) studied the antibody levels in breastmilk after inoculating mRNA vaccines (BNT162B2 and mRNA-1273) or viral vector vaccines (ChAdOx1-S). The study included 70 lactating women vaccinated with two doses of BNT162b2, 20 lactating women vaccinated with two doses of mRNA-1273 and 20 lactating women vaccinated with a single dose of ChAdOx1-S. The study found that IgA and anti-S1 IgG antibodies were produced in the breastmilk of all vaccinated mothers, while the level of IgA antibodies in the breastmilk of mothers immunized with BNT162b2 or mRNA-1273 was significantly higher than that of mothers vaccinated with ChAdOx1-S.

Calil V et al. (21) studied the antibody levels in breastmilk after inoculating inactivated vaccines (CoronaVac). They collected 170 samples from 20 lactating mothers who received two doses of the CoronaVac vaccine with an interval of 4 weeks. Some samples were taken before vaccination, other 7 samples per week were taken for three weeks after the second dose of vaccine, and breastmilk samples of 10 mothers were collected four months after the first dose of vaccine. Results showed that the level of anti-SARS-CoV-2 IgA increased in the first two weeks of the first injection, and increased significantly in the fifth and sixth weeks. In the seventh week, the level of specific IgA antibodies in breastmilk samples of 10 (10/20) mothers was higher than the serum transformation value. Compared to the breastmilk samples of four months after the first injection, the specific IgA antibodies level in the breastmilk samples of 5 mothers (5/10) was higher than the serum transformation value at that time.

Although some lactating women are vaccinated with mRNA vaccine, it is controversial whether nanoparticles or mRNA may enter breast tissue or transfer to breastmilk. The academy of breastfeeding medicine points out that this risk is low and can be degraded by the infant’s gastrointestinal system even if there is a possible small amount of mRNA (4). However, some believe that subclinical mastitis will lead to the destruction of the milk-blood barrier and the leakage of mRNA from the blood into milk (100). Infants having milk after vaccination have no adverse reactions within 28 days after ingestion (94), and specific antibodies can be detected in infant saliva and oral mucosa (11, 12). Therefore, after vaccination, mothers can transfer antibodies to infants to protect them from COVID-19 infection (101, 102).

At present, some studies have shown that breastmilk secretion of some mothers decreased after receiving the Moderna vaccine (103). Occasionally, some women also reported that the color of breastmilk changed into blue or blue-green after vaccination (99). Therefore, the mothers need to consult the doctor and choose whether to vaccinate according to their health situations.

### 5.2 Antibodies in the Human Milk of COVID-19 Patients and Convalescent Patients

Currently, a lot of researches have reported to detect specific antibodies for SARS-CoV-2 in the breastmilk of lactating mothers infected with COVID-19 (22) (**Table 1**).

Pace RM et al. (96) collected breastmilk samples from lactating mothers with confirmed COVID-19. The result showed that 76% of the samples contained SARS-CoV-2 specific IgA, 80% of the samples contained SARS-CoV-2 specific IgG and the concentration of immunoglobulins was much higher than that in breastmilk samples collected before the COVID-19 pandemic. In addition, 62% of the milk samples were able to neutralize SARS-CoV-2 infectivity *in vitro* (96). Pace RM et al. (82) collected breastmilk samples from 64 women with COVID-19, 75% of the breastmilk samples contained anti-RBD IgA and 77% of the breastmilk samples had IgA persisting for two months. In the study of Favara DM et al. (89), neutralizing antibodies in breastmilk remained positive for 6.5 months after infection. Bäuerl C et al. (97) collected breastmilk samples from 60 patients with COVID-19 and recovered patients, and it showed that 82.9% of the samples had at least one kind of antibody, 52.9% of the samples had IgM, IgG, and IgA antibodies. The positive rate of IgG continued to increase while IgA was relatively stable. Fox A et al. (98) collected breastmilk samples from eight recovered patients and seven suspected patients, and all samples presented significant specific IgA antibodies, while 80% of the samples showed anti-RBD IgA activity and 67% showed anti-RBD IgG and/or IgM activity.

Although specific antibodies were detected in the breastmilk of immunized, infected, or recovered individuals, it has been shown that breastmilk from lactating mothers infected with COVID-19 produces higher IgA, and breastmilk from lactating mothers after vaccination produces higher IgG, both of which have neutralization activity against live SARS-CoV-2 virus (22, 96, 104).

## 6 Conclusion

In summary, breastfeeding can reduce the risk of neonatal intestinal and respiratory infections and increase the survival rate of infants (1, 46). The risk of vertical transmission of SARS-CoV-2 through the breastmilk is low (33, 35, 105). Therefore, recommendations for pregnant COVID-19 patients have been rapidly shifting with various waves of the pandemic, but in general, World Health Organization (90), United Nations International Children’s Emergency Fund (106), and most countries (107, 108) support mothers with COVID-19 to contact and breastfeed their newborns and infants following the necessary hygiene rules, including disinfection with alcohol, wearing face masks, and obtaining written informed consent from parents (109).

Breastmilk can inhibit the activity of the SARS-CoV-2. Moreover, drugs (53, 75, 76) and microorganisms (77, 78) combined with LF can better inhibit the activity of SARS-CoV-2. In the future, the effect of other drugs which are capable of inhibiting SARS-CoV-2, such as artemether (110), artesunate (110), cepharanthine (111), and molnupiravir (112, 113), combined with human milk ingredients against SARS-CoV-2 can also be further investigated.

Specific antibodies with neutralizing activity against SARS-CoV-2 are produced in serum and breastmilk following maternal infection with SARS-CoV-2 (22) or immunization (18), while the antibodies can be transferred to the infants *via* the placenta or breastmilk, protect the infants from infection (92, 93). Even when the mother is too uncomfortable to breastfeed, the baby should be fed with expressed milk or pasteurized donated breastmilk in the human milk bank (114, 115).

Although there are multiple SARS-CoV-2 variants, including the omicron variant with immune escape ability (116, 117), vaccination can still provide satisfactory protection and reduces the severity of COVID-19 caused illness (118–120). Therefore, lactating mothers should be encouraged to vaccinate. In conclusion, lactating mothers infected with COVID-19 should be supported to breastfeed and encouraged to be vaccinated.

## Author Contributions

HHF, YGT, FTL, and LHS designed the research. ZHP, RLH, LLT, FXL, and YZC read and analyzed the papers. SQW, STH, SZZ, and XP An participated in the discussion. HHF, FTL, ZHP, RLH, LLT, FXL, and YZC wrote and revised the manuscript. All authors contributed to the article and approved the submitted version.

## Funding

This research was supported by National Natural Science Foundation of China (NO. 82151224), the Fundamental Research Funds for the Central Universities (BUCTRC201906, BUCTZY2022, China), Key Project of Beijing University of Chemical Technology (No. XK1803-06, China), National Key Research and Development Program of China (NO. 2022YFC0867500, 2019YFC1200502, 2020YFA0712102, BWS21J025, 20SWAQK22), Funds for First-class Discipline Construction (No. XK1805, XK2020-02 China), and H&H Global Research and Technology Center (grant no. H2021028).

## Conflict of Interest

The authors declare that the research was conducted in the absence of any commercial or financial relationships that could be construed as a potential conflict of interest.

## Publisher’s Note

All claims expressed in this article are solely those of the authors and do not necessarily represent those of their affiliated organizations, or those of the publisher, the editors and the reviewers. Any product that may be evaluated in this article, or claim that may be made by its manufacturer, is not guaranteed or endorsed by the publisher.
